# Highly Active NiRu/C Cathode Catalyst Synthesized by Displacement Reaction for Anion Exchange Membrane Water Electrolysis

**DOI:** 10.1002/smtd.202401179

**Published:** 2024-11-12

**Authors:** Stephan Ruck, Andreas Hutzler, Simon Thiele, Chuyen van Pham

**Affiliations:** ^1^ Helmholtz Institute Erlangen‐Nürnberg for Renewable Energy (IET‐2) Forschungszentrum Jülich GmbH Cauerstr. 1 91058 Erlangen Germany; ^2^ Department of Chemical and Biological Engineering Friedrich‐Alexander‐Universität Erlangen‐Nürnberg Cauerstr. 1 91058 Erlangen Germany

**Keywords:** anion exchange membrane, catalyst, membrane electrode assembly, nickel‐ruthenium, water electrolysis

## Abstract

Anion exchange membrane water electrolysis (AEMWE) is highly promising for cost‐effective green hydrogen production due to its basic operating conditions facilitating the use of non‐noble catalysts. While non‐noble Ni/Fe‐based catalysts are utilized at the anode, its cathode catalyst still requires precious Pt. Due to the high cost of Pt and the sluggish hydrogen evolution reaction (HER) at the cathode in basic conditions, developing alternative catalysts to replace Pt is highly important. Here, a synthesis procedure for a Ru‐based catalyst is reported and its high activity toward the HER in alkaline media is demonstrated in both half‐cell and single‐cell tests. The catalyst is synthesized in a two‐step approach. A highly dispersed Ni catalyst is prepared on carbon support in the first step. In the second step, Ru is deposited on its surface using a galvanic displacement reaction. The uniqueness of this method is that Ru is deposited over the entire electrically conductive surface, resulting in an isotropic and homogeneous Ru distribution within the catalyst powder. It is demonstrated that this material remarkably outperforms state‐of‐the‐art Pt/C catalysts in half‐cell and single‐cell tests. The single cell only requires 1.73 V at 1 A cm^−2^ with an overall PGM content of 0.05 mg cm^−2^.

## Graphical abstract

NiRu/C electrocatalyst for hydrogen evolution reaction in alkaline media is synthesized by a galvanic displacement method. Using this method, small clusters of Ru are homogeneously deposited on carbon support particles, resulting in superior Ru catalyst utilization. In anion exchange membrane electrolysis testing with a low overall PGM loading of 0.05 mg cm^−2^, the NiRu/C catalyst notably outperforms state‐of‐the‐art Pt/C.

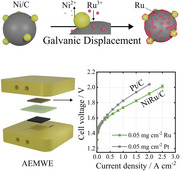

## 1 Introduction

Green hydrogen has gained wide attention over the last decade as a future energy carrier to substitute fossil fuel‐based energy systems. Green hydrogen is viably produced by water electrolysis using electrical energy from renewable sources like windmills or photovoltaics.^[^ ^1^, ^2^ ^]^ AEMWE is a comparably new technology for hydrogen production, offering several advantages compared to other electrolysis technologies.^[^ ^3^, ^4^, ^5^ ^]^ The alkaline environment of AEMWE allows the use of transition metals as catalyst materials instead of platinum group metals (PGM) such as Pt or Ir and non‐Ti materials for porous transport layers and flow fields.^[^ ^4^, ^6^, ^7^, ^8^, ^9^ ^]^ The quaternary ammonium group‐based ion exchange polymers used in AEMWE as membrane and electrode binder are less expensive and more environmentally compatible than the proton exchange polymer Nafion.

To establish AEMWE on an industrial scale, cost‐efficient catalysts must be developed with high operational performance in terms of activity and stability. NiFe‐based catalysts have been successfully developed to replace the scarce and costly Ir for the oxygen evolution reaction (OER) at the anode.^[^ ^10^ ^]^ NiFe‐layered double hydroxide catalysts (NiFe‐LDH) especially show high performance in membrane electrode assemblies (MEA).^[^ ^11^, ^12^ ^]^ For the cathode side of the AEMWE, carbon‐supported Pt (Pt/C) is still the catalyst of choice for the HER. Whereas the HER in acidic electrolytes is favorable with no significant overpotentials, the HER in alkaline conditions is two to three times more sluggish.^[^ ^13^ ^]^ To improve the catalytic activity of a Pt‐based catalyst in alkaline media, Wan et al.^[^ ^14^ ^]^ applied Ni(OH)_2_ shells showing exceptional activity. The HER in alkaline electrolytes follows a different mechanism, with the water‐splitting step being part of the reaction. This causes considerable overpotentials at the cathode, making the optimization of cathode catalysts more relevant to alkaline electrolysis.^[^ ^5^, ^15^, ^16^, ^17^ ^]^ In the mechanism of HER in alkaline solutions, adsorbed hydrogen (H_ad_) is the only reaction intermediate that occurs in both reaction pathways (Volmer–Heyrovsky; Volmer–Tafel, See Equations (1), (2), (3)), and thus its adsorption and removal strongly influence the reaction kinetics.^[^ ^18^ ^]^

(1) $$\mathrm{Volmer}:\;H_{2}O+e^{-}+*\to H_{ads}+OH^{-}$$

(2) $$\mathrm{Heyrovsky}:\;H_{ads}+H_{2}O+e^{-}\to H_{2}+OH^{-}+*$$

(3) $$\mathrm{Tafel}:\;2\;H_{ads\;}\to H_{2\;}+2*$$

where “*” denotes a free catalytic surface.

According to Sabatier's principle, a highly active catalyst's hydrogen binding energy (HBE) must not be too weak or too strong.^[^ ^19^, ^20^ ^]^ Using this principle, Xue et al.^[^ ^21^ ^]^ demonstrated that the HBE of NiRu catalysts is proper and thus showed high activity toward hydrogen oxidation reaction (HOR) in alkaline media. Numerous studies demonstrated high activity of complex structured Ru‐based catalysts, such as nanoclusters or single Ru atom catalysts for HER in alkaline media.^[^ ^21^, ^22^, ^23^, ^24^, ^25^, ^26^ ^]^

Zhu et al.^[^ ^27^ ^]^ investigated the role of Ru nanoclusters in alkaline HER and it was shown that Ru can significantly enhance the catalytic activity due to the accelerated ability of H_2_O‐bond breaking. It is known that the conditions of HER in a half‐cell three‐electrode setup (rotating disc electrode, RDE) significantly differ from those in the MEA, i.e., pH value, high reaction rates, and mass transport issue. The results from half‐cell measurements are not always transferable to single‐cell MEA tests. Thus, single cell measurements in MEA setups must be conducted to evaluate a catalyst fully.^[^ ^28^ ^]^

Given the promising alkaline HER activity of Ru and/or NiRu‐based catalysts proven previously in half‐cell tests, in this work, we develop a scalable synthesis method to obtain a highly dispersed carbon‐supported NiRu catalyst (NiRu/C) in sufficient amounts for single‐cell tests. We further demonstrate its applicability in AEMWE. Therefore, we utilize a two‐step synthesis to ensure homogeneous dispersion of Ni and Ru on the carbon supports. In the first step, well‐dispersed Ni nanoparticles on carbon support are developed. In the second step, Ru is introduced utilizing a galvanic displacement reaction. Using the displacement reaction, the amount of Ru can be precisely adjusted, and NiRu is homogeneously distributed over the entire surface of the carbon support. We demonstrate that NiRu/C catalysts perform superior to benchmark Pt/C reference when implemented as a cathode catalyst layer in MEA setup. The NiRu/C‐based cells achieve excellent performance of 1.73 V at 1 A cm^−2^ with an overall PGM loading of only 0.05 mg cm^2^.

## 2 Results and Discussion

### 2.1 Synthesis and Physical Characterization of NiRu/C Catalyst

**Figure 1:**
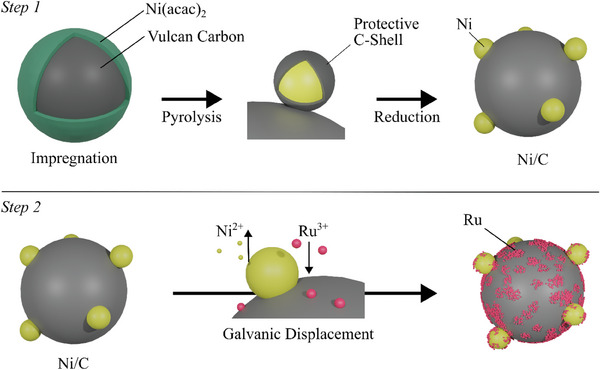
Schematic illustration of the two‐step synthesis approach used in this work. Step 1 is the synthesis of the Ni/C material (for structural analysis see Figure S1, Supporting Information), which includes the Ni(acac)_2_ wet impregnation, pyrolysis, and reduction process. During the pyrolysis, the protective carbon shell (see Figure S3, Supporting Information) is created to prevent particle sintering, and the Ni particles' reduction occurs when temperature increases and atmosphere is changed to H_2_/Ar. Step 2 contains the galvanic displacement of Ni with Ru, so Ru is deposited on the surface of the carbon support and Ni particles (see **Figure** **2**).

**Figure** **1** depicts the synthesis approach containing two steps. First, the carbon support was preconditioned in HNO_3_ to increase the amount of surface oxygen. This step increases the interaction and wettability of the carbon support material with the Ni^2+^ precursor in the consecutive impregnation step. Additional surface oxygen is essential for removing carbon shells (Figure S3, Supporting Information). The impregnation of the carbon support with Ni(acac)_2_ was adapted from Das et al.^[^ ^29^ ^]^ who initially showed the synthesis of Ni particles on SiO_x_ without large particle growth.^[^ ^29^, ^30^ ^]^

The structural analysis of the Ni/C pre‐catalyst was conducted using scanning electron microscopy (SEM) and high‐angle annular dark field scanning transmission electron microscopy (HAADF‐STEM) (Figure S1, Supporting Information). The Ni/C pre‐catalyst material was compared with a Ni/C reference catalyst synthesized by a standard wet impregnation and thermal reduction of NiCl_2_, denoted Ni/C_chloride_. SEM imaging shows that Ni/C_chloride_ contains large particles (up to 500 nm) in the bulk material, which are observed in carbon‐supported Ni catalysts from previous works and commercially available Ni/C materials, as well (Figure S2, Supporting Information).^[^ ^31^ ^]^ In contrast, the Ni/C pre‐catalyst using the Ni(acac)_2_ pyrolysis approach shows a homogeneous particle size distribution 23 ± 8 nm, with no large particles or agglomerates (Figure S1, Supporting Information).

The protective carbon layer, created in the pyrolysis step of the synthesis, successfully protected the Ni nuclei from agglomeration or Ostwald ripening. Changing the atmosphere in the furnace from N_2_ to Ar/H_2_ (5 vol.% H_2_) and increasing the temperature to 600 °C removes the carbon shell by consuming oxygen from the surface and from the pyrolyzed Ni(acac)_2_. When the carbon support was not pretreated with HNO_3_, the protective carbon shell was not (or not entirely) removed due to the lower amount of available oxygen (see Figure S3, Supporting Information, and the discussion in the caption).

We determined the Ni content of the resulting Ni/C material with thermogravimetric analysis (TGA) to be 23 ± 0.5 wt.%. (Figure S4, Supporting Information). The desired Ni content of 30 wt.% was not fully reached. A possible explanation could be that the precursor, which is not closely attached to the carbon support within the wet impregnation, is evaporated before the pyrolysis step (evaporation temperature of Ni(acac)_2_ = 220 °C).

In the second step, the Ni/C pre‐catalyst was dispersed in aqueous Ru^3+^ solution and was heated to 70 °C. The galvanic displacement occurred as Ru^3+^ is reduced by Ni to Ru and deposited on Ni/C according to Equations (4 and 5) to form NiRu/C. The galvanic displacement method can partially displace more active atoms with less active or more noble species. The Ru^3+^ ions with the higher oxidation potential can oxidize and displace the Ni metal with the lower oxidation potential.^[^ ^32^, ^33^, ^34^, ^35^ ^]^

(4) $$2\;\;Ru^{3+}+Ni\to 2\;\;Ru^{2+}+Ni^{2+}$$

(5) $$Ru^{2+}+Ni\to Ru+Ni^{2+}$$

**Figure 2:**
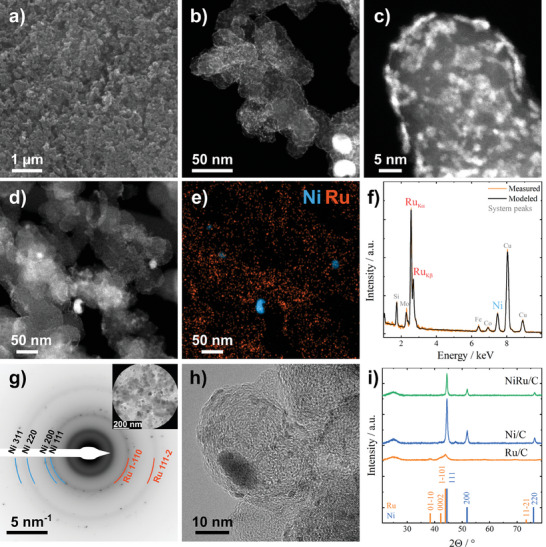
Physical characterization of NiRu/C catalyst. a) SEM image showing no large agglomeration in the bulk NiRu/C catalyst material. b) and c) overview and high‐resolution HAADF‐STEM images showcasing homogeneously distributed Ru on the carbon support surface (2.2 ± 0.9 nm). d) and e) HAADF‐STEM and corresponding STEM‐EDX spectrum image showing that Ru is distributed all over the carbon support and remaining Ni nanoparticles. f) integrated EDX spectrum showing Ni and Ru peaks. g) Selected area electron diffraction (SAED) pattern obtained from the region shown in the inset correlating with the nanostructure of the catalyst comprising sharp reflexes from Ni nanoparticles as well as diffuse diffraction rings originating from small Ru clusters and crystallites. h) High‐resolution bright‐field TEM image showing the layered, graphene‐like structure of the carbon support. i) XRD spectra of NiRu/C, Ni/C and Ru/C. Peaks corresponding to Ni 111 (lattice plane distance: 2.03 Å), 200 (1.77 Å), and 220 (1.24 Å).^[^ ^36^, ^37^ ^]^

The reaction supernatant was analyzed by ionic coupled plasma with optical emission spectroscopy (ICP‐OES) to determine the amount of deposited Ru. The result showed that Ru was fully consumed within 16 h of reaction time for each sample. TEM analyses of the NiRu/C samples (Figure 2) show that the displacement of Ni with Ru results in a Ru deposition on the carbon support and Ni surface. This indicates a direct Ru^3+^ reduction and thus deposition on the carbon support surface. Therefore, the participating electrons from Ni oxidation are conducted throughout the carbon support to reduce Ru^3+^ directly on the carbon support surface. This results in the deposition of homogeneously distributed small Ru clusters of 2.2 ± 0.9 nm over the entire surface of the carbon support particles (Figure 2b,c). These results indicate that Ru is deposited on all the electrically conductive surfaces but not favorably on Ni or Ru. STEM‐EDXS analysis supports these findings, which shows that Ru is deposited homogeneously over the carbon support surface (Figure 2d,f). The catalyst morphology is further characterized by powder X‐Ray diffraction (XRD) measurements of the NiRu/C catalyst compared to Ni/C pre‐catalyst and Ru/C (commercial catalyst) reference materials, as shown in Figure 2i. No signal of crystalline Ru is visible in the XRD spectrum of NiRu/C, pointing out that the deposited Ru species is either amorphous or too small to be detected by XRD. Furthermore, it was observed that metallic Ru was deposited across the entire surface of both carbon and Ni. Since it is not an intermixed Ni alloy, no significant electronic interaction between Ni and Ru was detected in our XRD analysis (Figure 2g,i).

The lattice structure of the crystalline Ni is visible and exhibits no peak shift compared to the Ni/C material before the displacement. Selected area electron diffraction (SAED) of the NiRu/C catalyst reveals the nanostructure of the catalyst showing sharp diffraction spots originating from fcc Ni, as well as two rather diffuse diffraction rings resulting from the small hexagonal Ru clusters and particles. Both rings of Ru largely overlap with reflexes from Ni and are, therefore, concealed in the XRD pattern. High‐resolution HAADF‐STEM (Figure 2c) shows the arrangement of Ru atoms in tiny clusters and crystallites on the surface of the crystalline carbon support structure (Figure 2h). Three different NiRu/C catalysts were synthesized with 2.5, 5.0, and 10.0 wt. % Ru, which was controlled by the RuCl_3_ precursor amount, since Ru^3+^ was fully consumed in the reaction.

### 2.2 Electrochemical Characterization

RDE measurements were conducted to initially evaluate the activity of the synthesized catalyst and quickly screen through different samples during synthesis development. This is to identify the promising samples for the more time‐consuming MEA tests later. To avoid the influence of mass transport as much as possible, the catalyst loading was kept constantly at a low value of 10 µg cm^−2^ (total catalyst mass) for every sample while ensuring a homogeneous coverage of the catalyst layer over the glassy carbon disc.

**Figure 3:**
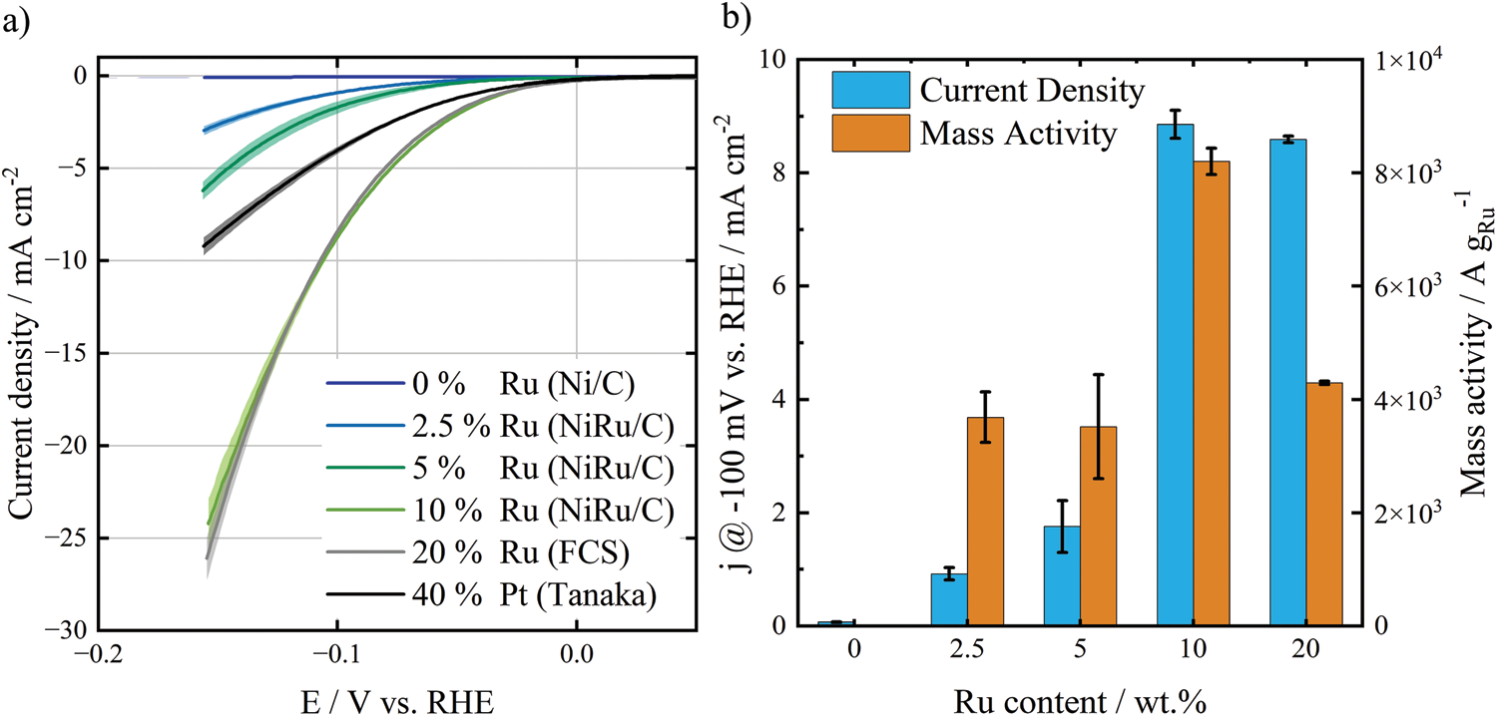
a) Linear sweep voltammetry curves of the synthesized NiRu/C catalysts with different Ru contents, compared to commercial reference materials: Ru/C (Fuel Cell Store) and Pt/C (Tanaka) tested in 1 m KOH at room temperature with constant overall catalyst loadings. b) Current density and calculated Ru‐mass activity at −100 mV versus RHE for NiRu/C with different Ru contents. The total catalyst loading for each material is 10 µg_catalyst_ cm^−2^. Shaded areas of the LSV curves exhibit the standard deviation of three separate measurements.

**Figure** **3** shows the HER activity of the catalysts in RDE measurements in alkaline media. It is shown that the HER activity of the NiRu/C catalysts increases with increasing Ru content. The Ni/C pre‐catalyst shows almost no activity toward HER within the potential window applied in this test. This indicates that the Ni particles in the NiRu/C catalysts do not contribute significantly to the overall activity and that Ru and Ru‐Ni interfaces are the active species of this catalyst, since Ni can enhance the HER activity by increasing proton availability.^[^ ^14^, ^38^ ^]^ Following the trend, the data show the NiRu/C sample with 10 wt.% Ru exhibits the highest activity. Figure S5 (Supporting Information) shows the Tafel plots of 10 wt.% NiRu/C compared to 40 wt.% Pt/C and 20 wt.% Ru/C reference materials, where Tafel slopes of 52.8, 67.7, and 58.7 mV dec^−1^ are determined, respectively. The optimal NiRu/C (10 wt.% Ru) exhibits similar activity to the commercial Ru/C catalyst containing 20 wt.% Ru, although the NiRu/C sample has only half of the Ru loading on the RDE tip compared to the Ru/C sample. This indicates that the Ru utilization of the NiRu/C catalyst is two times higher than that of commercial Ru/C. This can be explained by the unique structure of the NiRu/C catalyst, which contains extremely small Ru clusters homogeneously distributed over the carbon support surface (Figure 2c). Figure 3 shows the geometric current densities and Ru mass activity of the NiRu/C catalysts with different Ru contents. It indicates that the NiRu/C catalyst with 10 wt. % Ru is optimal, exhibiting the highest Ru‐mass activity and geometric current density.

**Figure 4:**
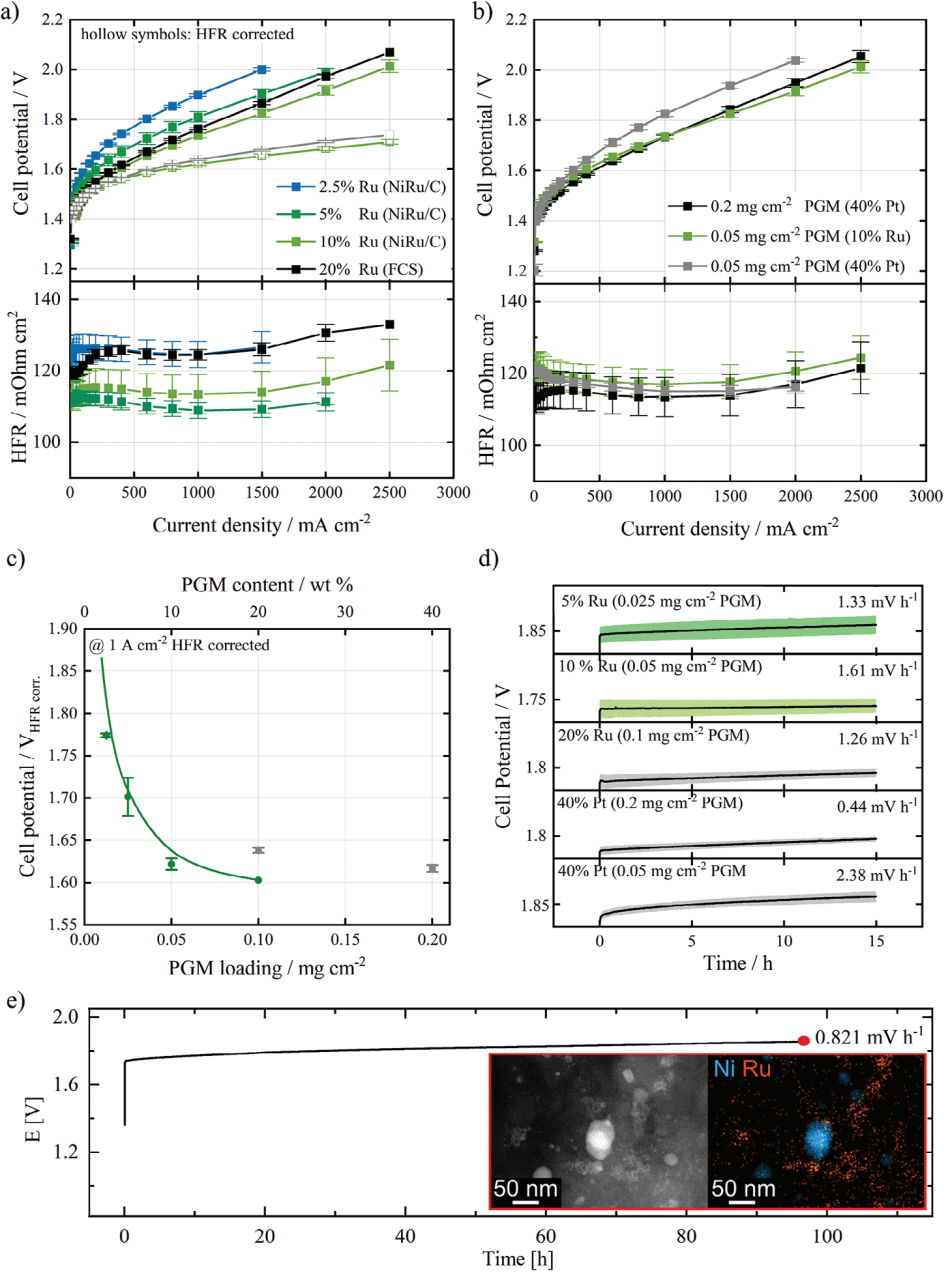
MEA performance in AEMWE single‐cell experiments with different catalysts. a) Polarization curves of the synthesized NiRu/C catalysts with different Ru contents compared to the commercial Ru reference (20 wt.% Ru, Fuel Cell Store) with constant catalyst loading of 0.5 mg_catalyst_ cm^−2^. b) Polarization curves of the synthesized 10 wt.% Ru, NiRu/C catalyst compared to state‐of‐the‐art Pt/C (40 wt.% Pt, Tanaka) catalysts with same catalyst loading (0.2 mg_Pt_ cm^−2 ^ =  4fold PGM loading) and same PGM loading (0.05 mg_PGM_ cm^−2^). c) HFR corrected cell potentials at 1 A cm^−2^ over PGM contents (0–20 wt.% Ru) of NiRu/C (green), 20 wt.% Ru/C reference and 40 wt.% Pt/C reference (gray) in the electrode. d) Stability tests over 15 h constant current at 1 A cm^2^. e) 100 h durability test based on constant current at 1 A cm^2^.The inset shows end‐of‐test TEM images of the catalyst structure. Error bars (a–c) and shaded area (d) exhibit the standard deviation of three separate measurements.

**Figure** **4** shows the MEA performance in full‐cell tests of the studied catalyst materials. For testing the MEA performance, the cathodes were fabricated with low catalyst loadings to ensure that anode side limitations would not interfere with the effects of cathode side catalysts. Therefore, an overall catalyst loading (carbon + metals) of 0.5 mg_catalyst_ cm^−2^ was used for all electrodes to ensure similar morphology (except the PGM normalized Pt/C reference). SEM analysis of the electrode's morphology is shown in Figures S6 and S7 (Supporting Information). The results show that the structure of all tested electrodes is comparable, and differences in mass transport phenomena are assumed to be negligible.

Further, the specific surface area was compared using N_2_ adsorption/desorption and the Brunauer–Emmett–Teller (BET) method. The BET surface area slightly increases during the Ni/Ru displacement from 163 to 181 m^2^ g^−1^ for Ni/C and NiRu/C, respectively. This increase can be addressed by the Ru deposition on the carbon support. The reference material Pt/C has a BET surface area of 173 m^2^ g^−1^, which leads to the assumption that the porous carbon support material mainly defines the BET surface area and, thus, is comparable for all tested catalysts and reference materials (Figure S8, Supporting Information).

Figure 4a show that the MEA performance of the catalysts with different Ru contents follows the trend, which was consistent with the RDE experiment results. The catalyst's performance increases with increasing Ru contents up to 10 wt.%. Further increasing beyond this does not result in a significant performance increase. Additionally, a sample with 20 wt.% Ru was prepared with the galvanic displacement method. The result is shown in Figure 4c, which shows just a slight increase of performance compared to the 10 wt.% sample. The 20 wt.% Ru (displacement) sample outperforms the 20 wt.% Ru (FCS) sample, which underlines the structural advantage of the synthesis method (See Figure S9, Supporting Information). Two experiments were conducted to fairly compare the performance of NiRu/C catalysts to state‐of‐the‐art Pt/C catalysts (Figure 4b). In one experiment, the total catalyst mass in the electrode was kept constant, in the other experiment, the catalyst loading of the Pt/C reference was adjusted to the same PGM loading as NiRu/C of 0.05 mg_PGM_ cm^−2^ to evaluate the overall cell performance. The data shows that the 10 wt.% Ru, NiRu/C catalyst performed similarly in the MEA to Pt/C reference, while the NiRu/C base MEA used four times less PGM loading. When the PGM loading was kept constant, the 10 wt.% Ru, NiRu/C catalyst notably outperformed Pt/C reference. With the optimal NiRu/C catalyst (10 wt.% Ru), the MEA loaded with an overall PGM content of 0.05 mg_PGM_ cm^−2^ (for both cathode and anode) exhibited a cell voltage of 1.73 V at 1 A cm^−2^, which is significantly lower than that of 1.83 V for Pt/C reference at the same PGM loading (Figure 4b). This underlines the exceptional PGM utilization of NiRu/C catalyst synthesized by the galvanic displacement method. The Ni/C pre‐catalyst was also tested in MEA (Figure S10, Supporting Information) and showed poor performance, as expected from the RDE results.

The results again confirm the difficulty of the aforementioned transferability of RDE results to MEA performance. The activity trend observed by RDE within the Ru‐based catalysts is shown to be transferred into the MEA, but the performance of the Pt/C samples is much higher in the MEA than assumed by the RDE. In this context, we note the impact of the anode in single‐cell tests. While in RDE, an electrode gets polarized with defined overpotentials, and the catalyst layer is always operated at its limit, the processes occurring in MEA are much more complex due to the influence of the other electrode. In other words, there is a limit to how much the cathode can improve the MEA performance which is the point where the anode becomes limiting. This is clearly shown in Figure 4c, where PGM contents increased from 10 wt.% to 20 wt.% do not significantly improve the cell's performance any further because the anode side limits the cell's performance. We show that the cathode side can fulfill its functionality to sustain the anode activity with a minimum Ru loading of 0.05 mg_Ru_cm^−2^ using the NiRu/C catalyst of our synthesis method. Figure 4d shows the 15 h constant current hold experiments. 15 h of constant current hold is not enough to evaluate the long‐term stability of the catalyst. Nevertheless, it can be utilized to evaluate if a material undergoes different degradation mechanisms than the reference materials which would result in different degradation rates. To evaluate the structural stability of the catalyst, a 100 h constant current hold was performed, which showed that the fine Ru structure remained largely unchanged (Figure 4e). The cell showed an overall degradation rate of 0.821 mV h^−1^, half of the rate shown by the 15 h test. This indicates that the cell gets stabilized over long‐term operation. The low degradation of the high‐loading Pt/C electrode compared to the low‐loading Pt/C electrode indicates that the catalyst loading in the electrode significantly affects the degradation. The cells with 0.05 mg_Ru_ cm^−2^ NiRu/C (10 wt.% Ru) showed a degradation rate of 1.61 mV h^−1^, which is lower than that of 2.38 mV h^−1^ for Pt/C‐based cells with the same loading but higher than 0.44 mV h^−1^ for the high Pt/C loading cells. The degradation rates of the synthesized NiRu/C catalysts are quite comparable with the commercial Ru/C catalyst.

The phenomena discussed before do affect degradation as well. Since the catalyst layer with high Pt/C loading could not be fully utilized, it can degrade but still hold the performance at the cathode side limit. Degradation mechanisms in AEMWE are very complex and not fully understood at this point. Ion exchange polymers suffer from instability under an alkaline environment and can cause additional degradation by detachment of the catalyst. Therefore, a full investigation of the observed degradation mechanisms is not in the scope of this work.^[^ ^6^, ^13^ ^]^

To our knowledge, our cell performance with such a low overall PGM loading is superior to previous reports, as shown in **Table** **1**, which gives an overview of MEA cell performance based on literature. Since the membrane materials used significantly influence the cell performance, we only compare our cell's performance with previous works that used the same membrane material (Aemion).

**Table 1:** Overview of AEM‐WE performances using Aemion membrane and different catalysts.

Work	Anode catalyst (loading [mg_cat_ cm^−2^])	Cathode catalyst (loading [mg_PGM_ cm^−2^])	Membrane (thickness [µm])	Electrolyte (temperature [°C])	MEA performance [V @ 1 | 2 A cm^−2^]
This work	NiFe‐LDH (2)	NiRu/C (0.05)	Aemion AF3‐HWK9‐75 (75)	1 m KOH (70)	1.73 | 1.91
This work	NiFe‐LDH (2)	Pt/C (0.05)	Aemion AF3‐HWK9‐75 (75)	1 m KOH (70)	1.83 | 2.04
This work	NiFe‐LDH (2)	Pt/C (0.2)	Aemion AF3‐HWK9‐75 (75)	1 m KOH (70)	1.73 | 1.95
Hager et al. ^[^ ^12^ ^]^	NiFe‐LDH (2)	Pt/C (0.5)	Aemion AF3‐HWK9‐75 (75)	1 m KOH (70)	1.71 | 1.91
Ruck et al.^[^ ^31^ ^]^	IrOx (1.5)	Pt/C (0.1)	Aemion AF1‐HNN8‐50 (50)	1 m KOH (70)	1.79 | 2.0
Koch et al.^[^ ^39^ ^]^	IrOx (1)	Pt/C (0.5)	Aemion AF2‐HLE7‐25 (25)	0.1 m KOH (60)	1.95 | –
Fontin et al.^[^ ^40^ ^]^	Ir black (3.8)	Pt/C (1)	Aemion AF1‐HNN8‐50 (50)	1 m KOH (50)	1.8 | 2.0
Gonzalez et al.^[^ ^41^ ^]^	IrOx (2)	Pt/C (1)	Aemion AF2‐HWP8‐75	1 m KOH (80)	1.71 | 1.9

## 3 Conclusion

In this work, we successfully synthesized a homogeneous and highly dispersed Ni‐based electrocatalyst supported on Vulcan carbon by using Ni(acac)_2_ precursor pyrolysis to form a protective carbon shell, which prevents particle growth. This Ni/C pre‐catalyst is then used in a consecutive galvanic displacement step of Ni by Ru to deposit Ru on the surface of Ni/C to form NiRu/C catalysts. The uniqueness of this mechanism is that the electrons participating in the redox reaction (Ni/Ru galvanic displacement) are conducted through the carbon support to reduce the Ru^3+^ precursor. This allows Ru to deposit over the entire surface of the carbon support, forming Ru clusters of 2.2 ± 0.9 nm, and results in a very homogeneous distribution of Ru over the porous support material.

The NiRu/C catalysts are electrochemically characterized in RDE and single‐cell MEA tests. In the RDE experiments, NiRu/C showed very high activity toward the HER in alkaline media. We demonstrate that utilizing only 10 wt.% Ru in the displaced NiRu/C catalyst achieves similar activity to a commercial reference containing 20 wt.% Ru (twofold Ru loading on electrode) or 40 wt.% Pt (fourfold PGM loading on electrode) on a similar carbon support.

Three NiRu/C catalysts with 2.5, 5.0 and 10.0 wt.% Ru were synthesized and tested in MEA single‐cell experiments and compared to commercial 20 wt.% Ru/C and 40 wt.% Pt/C reference catalysts. The results show that our catalysts notably outperformed the state‐of‐the‐art Pt/C (40 wt.%, Tanaka) with the same PGM loading, and reached similar performance with four times less PGM loading. The cell achieved 1 A cm^−2^ at 1.73 V with an overall PGM loading of 0.05 mg_PGM_ cm^−2^.

All MEA tests included a 15 h constant current hold to evaluate the stability of each catalyst. The results show that the synthesized NiRu/C catalyst does not exhibit a degradation behavior different from that of commercial reference catalysts. All cell degradation is most likely due to the detachment of catalyst layer fragments caused by ionomer degradation rather than catalyst dissolution. The structural stability of the catalyst was investigated after a 100 h constant current hold experiment. No notable change with only little agglomeration of Ru clusters was observed at the end‐of‐test sample.

## 4 Experimental Section

### Synthesis

The NiRu/C catalysts are synthesized in two steps to ensure a homogeneous dispersion of Ni and Ru on the carbon supports. In the first step, well‐dispersed Ni nanoparticles on carbon support (Ni/C pre‐catalyst) are developed. In the second step, Ru is introduced using galvanic displacement.

### Synthesis—‐Synthesis of Ni/C Pre‐Catalyst

This synthesis was adapted from Das et al.,^[^ ^28^ ^]^ who successfully developed the synthesis on silica supports.

As a first step, the Vulcan Carbon XC‐72 support was pretreated with 30% HNO_3_ for 180 min at 80 °C to increase the amount of surface oxygen.^[^ ^29^ ^]^

For the synthesis of Ni/C pre‐catalyst, 0.35 g of pretreated Vulcan Carbon XC‐72R were dispersed in a 100 mL round bottom flask containing a mixture of 30 mL ethanol (Sigma–Aldrich, 99.9%) and 10 mL toluene (Sigma–Aldrich, 99.9%) for 1 h in an ultrasonication bath followed by 10 min using an ultrasonication horn. Afterward, 0.691 g of Ni(acac)_2_ (nickel(II)acetylacetonate, Sigma–Aldrich, 95%) was added, and the mixture was stirred for 48 h at room temperature (RT). The wet impregnation was carried out by evaporating the solvent using a rotary evaporator and drying at 20 mbar for 30 min afterward. The sample was then collected and heated to 270 °C for 1 h under N_2_ atmosphere in a tube furnace. The pyrolysis of the acetylacetonate framework is taking place at another holding point at 450 °C. Afterward, the atmosphere was changed to H_2_/Ar (5% H_2_ in Ar, 150 mL min^−1^), the temperature was increased to 600 °C, and the temperature was held for 2 h before cooling down. The Ni content of the catalyst was then determined by thermogravimetric analysis (TGA) to be 23.5 ±0.5 wt.% Ni (Figure S4, Supporting Information). Ni/C (40 wt.%, Fuel Cell Store) was used as reference material.

### Synthesis—Synthesis of NiRu/C by Ni/Ru Galvanic Displacement

In the galvanic displacement step, 100 mg of the synthesized Ni/C pre‐catalyst was dispersed in 30 mL H_2_O (MilliQ, Merck Millipore) in a 50 mL round bottom flask for 15 min in an ultrasonication bath. Afterward, calculated amounts of RuCl_3_ (Reagent Plus, Sigma–Aldrich, 42% Ru) were added, and the mixture was stirred for 16 h at 70 °C. Under this condition, Ni was displaced with Ru^3+^, and Ru metal was deposited on Ni/C to form NiRu/C catalyst. Then, the NiRu/C catalyst was filtrated and washed with 150 mL of deionized water.

### Physical Characterization

For structural analysis, TEM was carried out using a Talos F200i (Thermo Fisher Scientific) equipped with a Schottky field‐emission gun (X‐FEG) and a Dual Bruker XFlash 6 | 100 EDXS detector. A Thermo Fisher Scientific low background, high visibility double‐tilt holder with a Mo clamp was utilized for imaging. For spectrum imaging and (scanning) transmission electron microscopy ((S)TEM), a primary electron energy of 200 keV was used. For STEM the electron probe was tuned to a beam current of 41 pA and a convergence angle of 10.5 mrad. A HAADF detector was employed for collecting elastically scattered electrons toward an angular range of 58–200 mrad. For SAED an aperture with a diameter of 40 µm was employed. Catalyst samples were dispensed on conventional lacey carbon Cu grids and were plasma cleaned before imaging using a Tergeo‐EM Plasma Cleaner (PIE Scientific).

A scanning electron microscope (Vega3 TEscan) was used for structural analysis. Therefore, the catalyst powder was fixed on a SEM stub with a conductive double‐sided adhesive carbon pad. Surface imaging was performed at an accelerating voltage of 20 kV using a secondary electron detector.

The supernatant of the Ni/Ru displacement reaction was analyzed by ionic coupled plasma with optical emission spectroscopy (ICP‐OES, PerkinElmer Optima 8300 DV) to determine the Ru^3+^ concentration in the reaction supernatant.

XRD patterns were recorded using a Bruker D8 advanced diffractometer, while applying Cu‐Kα radiation with a wavelength of 0.154 nm and using an angular step size of 0.02° 2Θ within an angular range of 15–90°.

Furthermore, N_2_ adsorption‐desorption isotherms were recorded at 196 °C (micromeritics TriStar II PLUS) with a micromeritics (VacPrep 061) sample degas system. The specific surface area was calculated using the Brunauer–Emmett–Teller (BET) method.

### Electrochemical Characterization—Rotating Disc Electrode (RDE)

The electrochemical activity of catalysts is determined by rotating disk electrode experiments.

Therefore, an RDE was set up, and RDE glassy carbon tips (Pine Research) were used. All experiments were conducted using a Biologic SMP300 potentiostat. The catalyst inks were prepared by mixing 1.92 mg of the corresponding catalyst powder with 5 mL 1‐propanol (Sigma–Aldrich, 99.9%) and 5 µL of Nafion D520 solution (Chermours). In the following, the ink was dispersed for 15 min using an ultrasonic horn (UP200 St, Hielscher Ultrasonics GmbH with a S26d7 sonotrode). Five microliters of the catalyst ink was drop cast onto the glassy carbon tips and dried at room temperature, which resulted in a catalyst loading of 10 µg cm^−2^ (metal + carbon) and an ionomer content of ≈10 wt.%. All RDE experiments were conducted in an in‐house designed PTFE cell with 1 m potassium hydroxide (KOH, VWR, AnalR NORMAPUR, 88.4%) as the electrolyte, which was degassed for a minimum of 30 min with Ar (99.999 mol%, AirLiquide Deutschland GmbH) before each experiment. A graphite rod and a reversible hydrogen electrode (RHE, gaskatel HydroFlex) were used as a counter and reference electrode, respectively.

### Electrochemical Characterization—Membrane Electrode Assembly (MEA)

In this study, a catalyst‐coated substrate approach (CCS) was used as electrode fabrication method. For this, a spray coater (Sonotek, ExactaCoat) with an ultrasonic nozzle (AccuMist, 48 kHz) was used. The spraying parameters varied depending on the electrode substrate material and are listed in Table S1 (Supporting Information). In this study, a nickel porous transport layer (PTL, BEKPOR 2N1180.25, Bekaert) was used as the anode substrate, and a carbon gas diffusion layer (GDL, Freudenberg, H23C2) was used as the cathode substrate.

The fabrication process and catalyst loading for all anode and cathode electrodes were kept constant throughout this study. For anode fabrication, a catalyst ink was prepared by mixing calculated amounts of NiFe layered double hydroxide (Matteco) with the appropriate amounts of water and stirring overnight. Afterward, calculated amounts of ethanol (EtOH:H_2_O = 1:1) and Nafion D520 were added to the mixture to result in 3 wt.% solids and 10 wt.% binder content. The ink was ultrasonicated (UP200 St, Hielscher Ultrasonics GmbH) with a S26d7 sonotrode for 60 min at 40 W while being cooled in an ice bath before spray coating. The nickel PTLs were laser cut into squares of 5 cm^2^ and fixed onto the heating plate during spray coating using magnetic tape. Anode electrodes were spray‐coated with a catalyst loading of 2 mg_NiFeLDH_ cm^2^. For cathode electrodes, an ink with 1 wt.% solid was prepared containing 10 wt.% binder and EtOH+H_2_O (1:1) as solvent. The ink was mixed, stirred overnight, and ultrasonicated, similar to the anode ink, before spray coating. Cathodes are fabricated with a loading of 0.5 mg_catalyst _cm^−2^ (metal + carbon), with the PGM normalized Pt/C reference as an exception, where the loading was set to 0.05 mg_Pt_ cm^−2^.

For spraying catalyst ink on GDLs, squares of 36 cm^2^ were punched out and fixed onto the spray coater's heating plate. 5 cm^2^ squares were punched out as electrodes for MEA testing after spraying. The ink in the syringe of the spray coater was continuously stirred to prevent sedimentation of catalyst particles during the process.

To ensure the desired catalyst loading and a constant catalyst deposition rate, a reference 5 cm^2^ square was weighed before, during, and after spray coating using a Sartorius Cubis microscale (MSA66S000DH).

### Electrochemical Characterization—Single Cell Testing

For AEMWE testing, a membrane (Aemion, AF3‐HWK9‐75, Ionomr) was preconditioned (24 h in 1 m NaCl, 48 h in 1 m KOH) and sandwiched in between the fabricated electrodes. The anode was connected as a working electrode and the cathode as a counter/reference electrode, respectively. For the electrochemical characterization of MEAs in the AEMWE, the cell rested (open circuit potential) for 90 min to heat up the cell. Afterward, constant current (CstC) at 5 A for 120 min was performed as a break‐in to reach a stable operation. Lastly, a polarization curve was recorded galvanostatically with a 3‐min holding time at every current step. The first current holds were performed at 10, 50, and 100 mA. In the range of 100 to 500 mA, the currents were increased by 100 mA steps. In the range of 1 to 10 A, the step size was increased to 1 A. Finally, currents were held at 12.5 and 15 A. The last 30 s of each current hold were averaged to obtain a data point. After each holding point, GEIS measurements were performed in the frequency range between 100 and 1 Hz. After the polarization curve, the cell was held at a constant current of 5 A for 15 h. For the 100 h test, a constant current (CstC) at 5 A for 120 min was performed as a break‐in and another CstC for 98 h.

## Conflict of Interest

The authors declare no conflict of interest.

## Author Contributions

S.R. performed conceptualization, data curation, formal analysis, investigation, methodology, validation, visualization, writing – original draft, writing –review & editing. A.H. performed conceptualization, formal analysis, investigation, supervision, writing – review & editing. S.T. performed conceptualization, acquired funding acquisition, project administration, resources, supervision, writing – review & editing. C.V.P. performed conceptualization, funding acquisition, supervision, writing – review & editing.

## Supporting information



Supporting Information

## Data Availability

The data that support the findings of this study are available in the supplementary material of this article.
