# Supplementary material for: Highly Active NiRu/C Cathode Catalyst Synthesized by Displacement Reaction for Anion Exchange Membrane Water Electrolysis
Source: Small Methods. 2024 Nov 12;9(4):2401179. doi: 10.1002/smtd.202401179 (PMC12020336; doi:10.1002/smtd.202401179)
Supplement: Supplementary file 1 — Supporting Information [file SMTD-9-2401179-s001.docx]

Supporting Information

Highly active NiRu/C cathode catalyst synthesized by displacement reaction for anion exchange membrane water electrolysis

Stephan Ruck*, Andreas Hutzler, Simon Thiele, Chuyen van Pham*

**Table S1**. Spray coating parameter

| Parameter | PTL (Anode) | GDL (Cathode) |
| --- | --- | --- |
| Flow rate / ml min^-1^ | 0.45 | 0.33 |
| Nozzle speed / mm s^-1^ | 170 | 140 |
| Heating plate temperature / °C | 120 | 100 |
| Nozzle Height / mm | 37 | 37 |
| Shaping Air / kPa | 0.6 | 0.6 |
| Pitch / mm | 1.5 | 1.5 |
| Ultrasonication power / W | 4.5 | 4.5 |


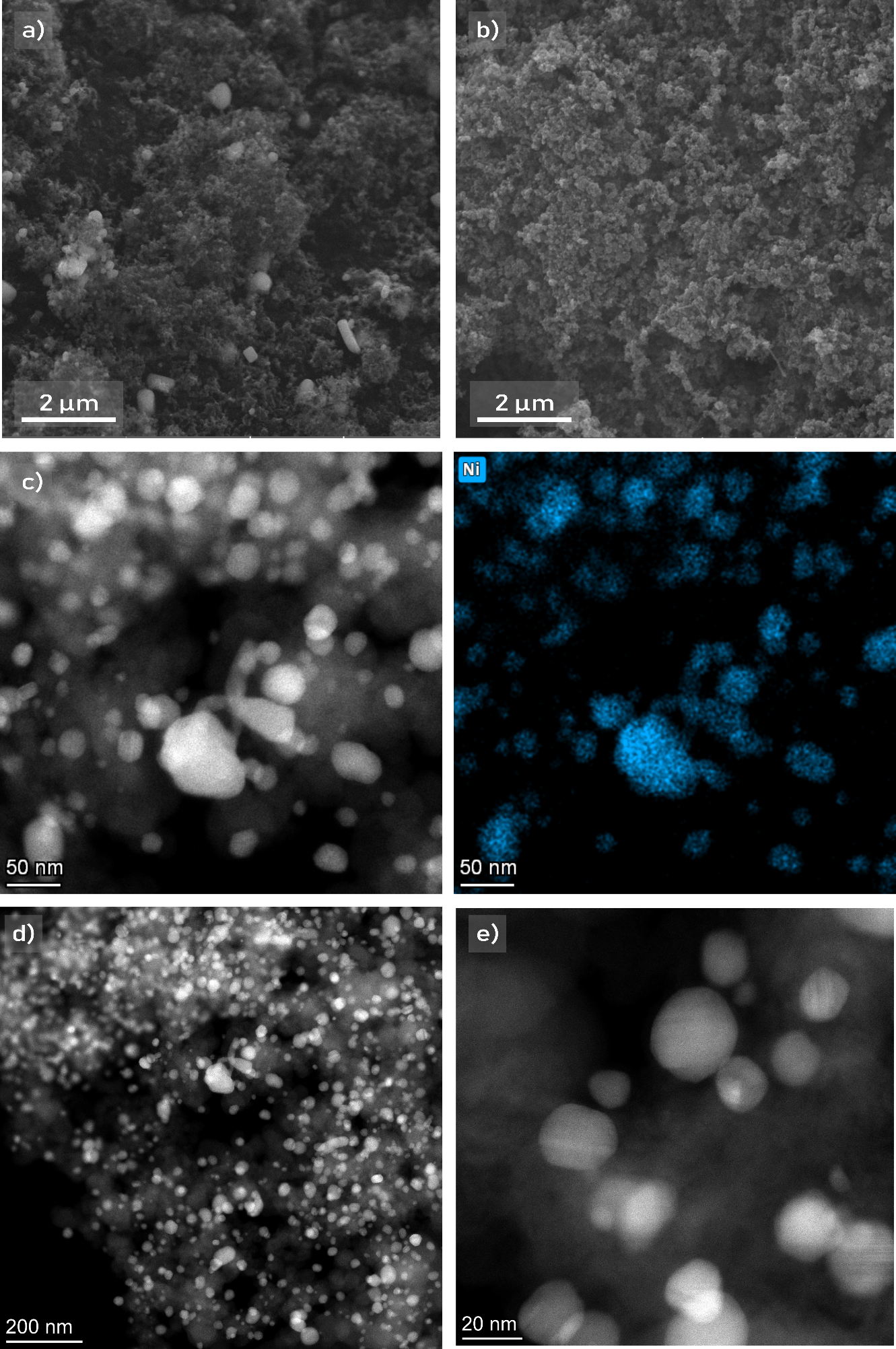


**Figure S1:** Structural analysis of step 1 Ni/C catalyst. a) SEM image of Ni/C_chloride_ catalyst synthesized by a standard wet impregnation and thermal reduction of NiCl_2_. Large Ni particles of up to 500 nm are visible in the bulk material. b) SEM image of Ni/C catalyst synthesized by incorporating Ni(acac)2 pyrolysis. The SEM images show no large Ni particles in the bulk material, indicating a successful application of the protective carbon shell by the pyrolysis of Ni(acac)_2_ c) STEM-EDXS analysis of Ni/C catalyst. d-e) HAADF-STEM images of Ni/C catalyst, with a homogeneous particle size distribution 23± 8 nm (200 particles measured in d))


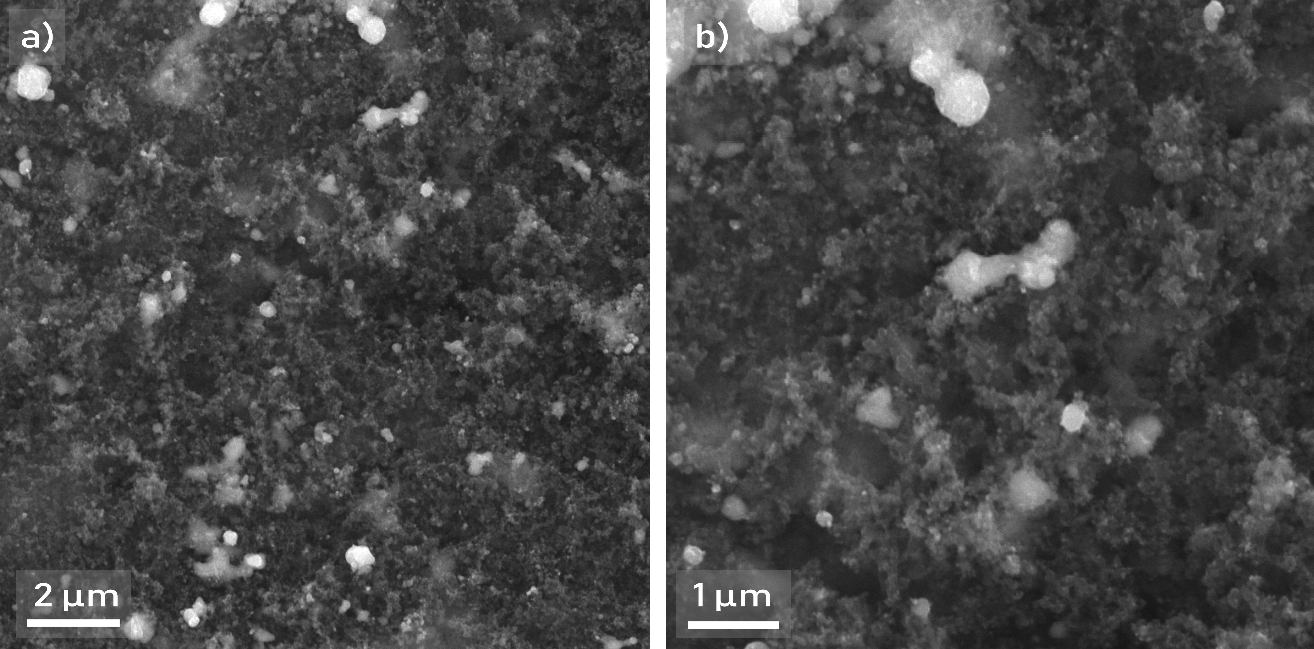


**Figure S2:** Structural analysis of commercial Ni/C (40 wt.% Ni) catalyst. Large Ni particles of up to 500 nm are visible in the bulk material.


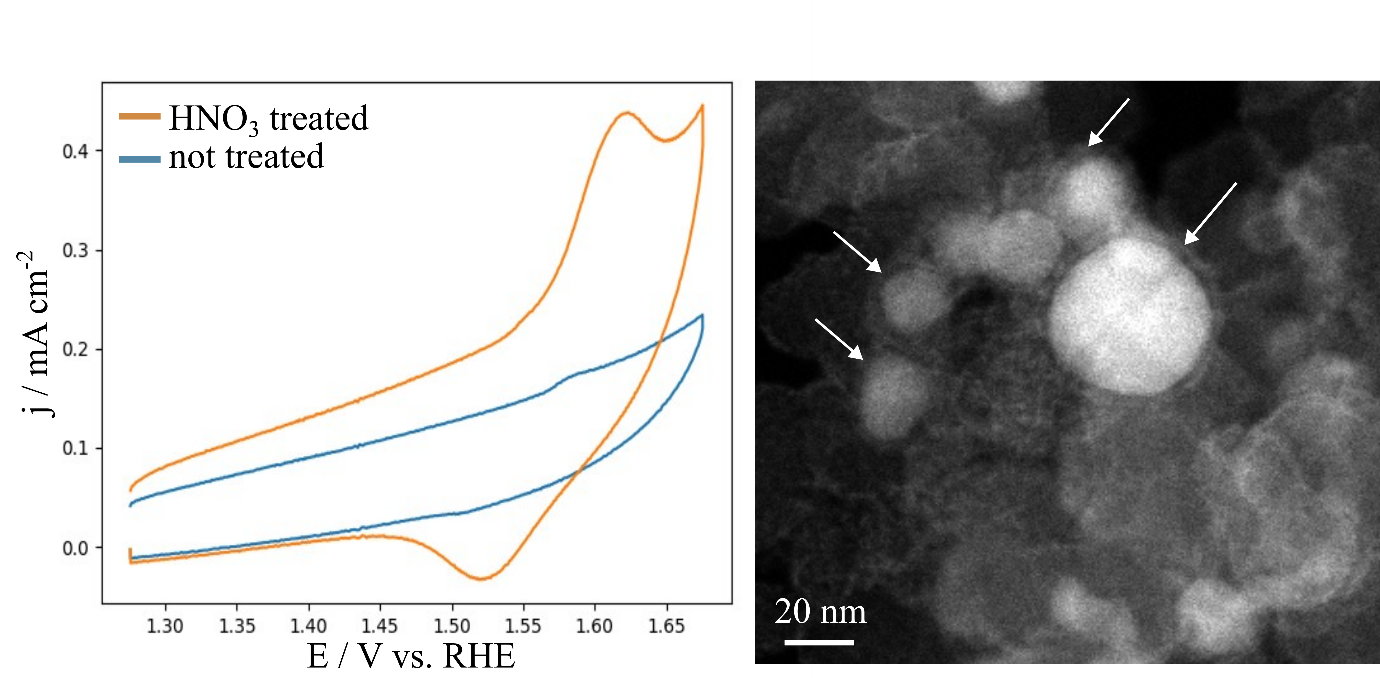


**Figure S3**: Analysis of the protective carbon shell and influence of HNO_3_ pre-treatment. (right) Cyclic voltammetry shows that the Ni oxidation and reduction peaks are clearly visible for the HNO_3_-treated sample compared to the untreated sample, which is caused by the remaining protective carbon shell, which is not fully removed in the untreated sample. (left) The HAADF-STEM analysis underlines that the removal of the protective carbon shell strongly depends on the surface oxygen of the carbon support material. When carrying out the Ni Ru displacement in the second step of the synthesis, Ru is not fully consumed by the reaction due to the carbon protected Ni particles. Some particles are still participating in the reaction and Ru is deposited and top of the carbon shell.


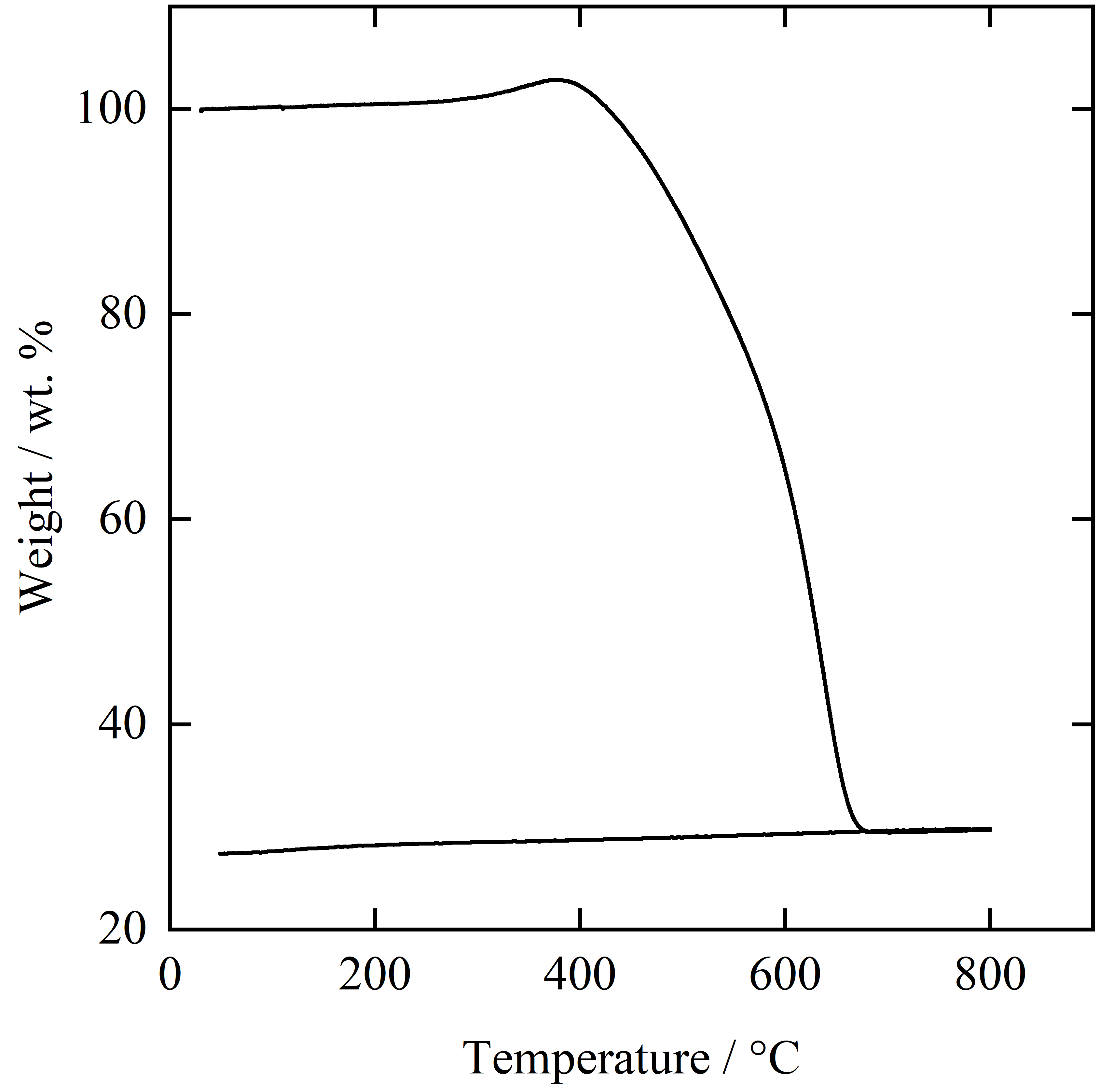


**Figure S4**: Thermogravimetric analysis (TGA) of the synthesized Ni/C catalyst under synthetic air. Ni gets oxidized to NiO at ~ 400 °C, and carbon oxidation over 400 °C. The Ni content in the catalyst can be calculated, assuming just NiO is left.^[1]^


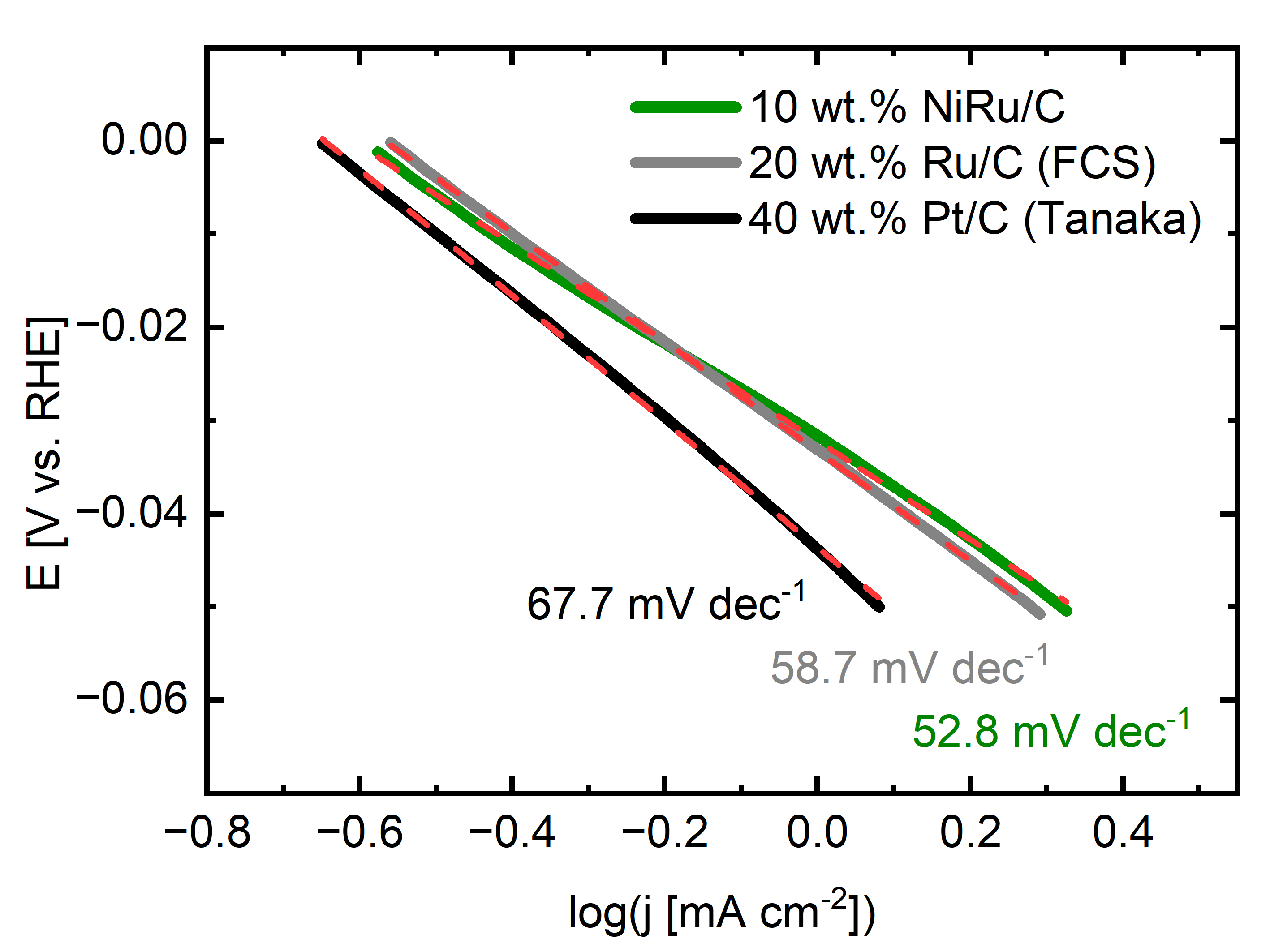


**Figure S5:** Tafel analysis for 10 wt.% NiRu/C compared to 40 wt.% Pt/C (Tanaka) and 20 wt.% Ru/C reference materials, where Tafel slopes of 52.8 mV dec^-1^, 67.7 mV dec^-1^ and 58.7 mV dec^-1^ are determined, respectively.


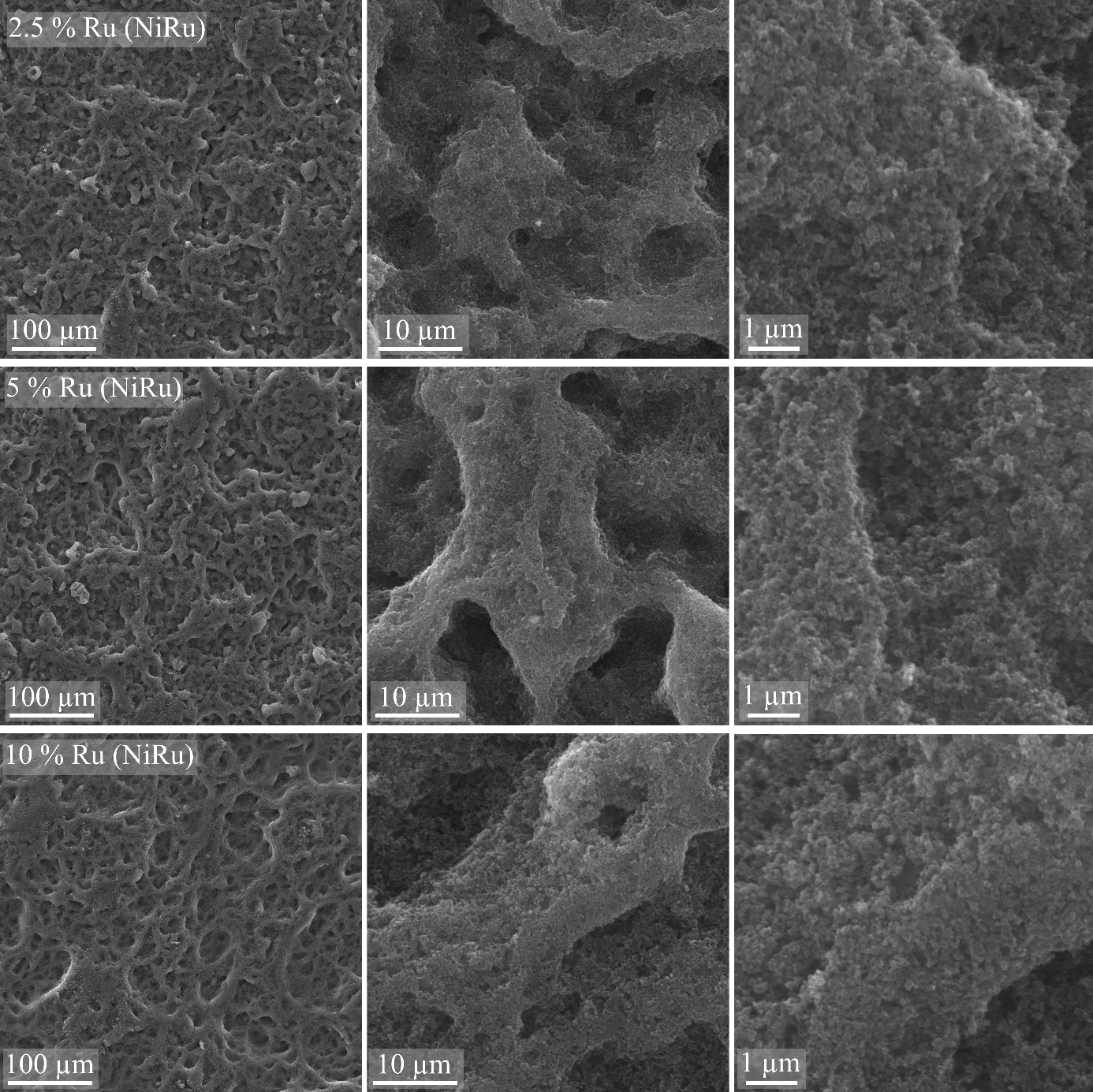


**Figure S6:** SEM images of NiRu/C cathode electrodes. The structural analysis shows similar structures on all scales. The catalyst loading in all electrodes is constant with 0.5 mg_cat_ cm^-2^:


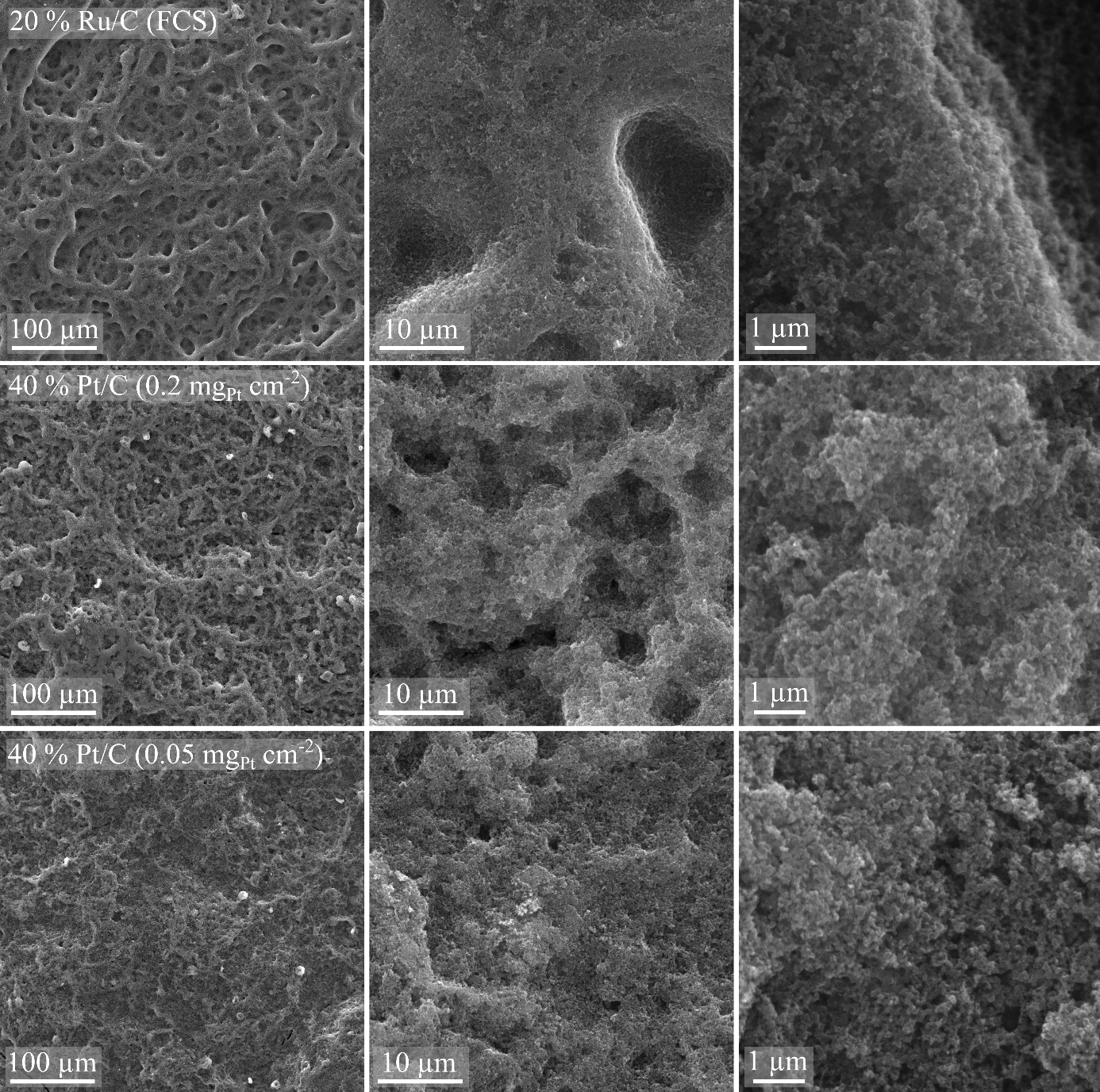


**Figure S7:** SEM images of reference cathode electrodes using commercial PGM catalysts. The loading is constant with 0.5 mg_cat_ cm^-2^ except for the 0.05 mg_Pt_ cm^-2^ sample which was chosen to match the PGM content of the 10 wt.% NiRu/C sample.


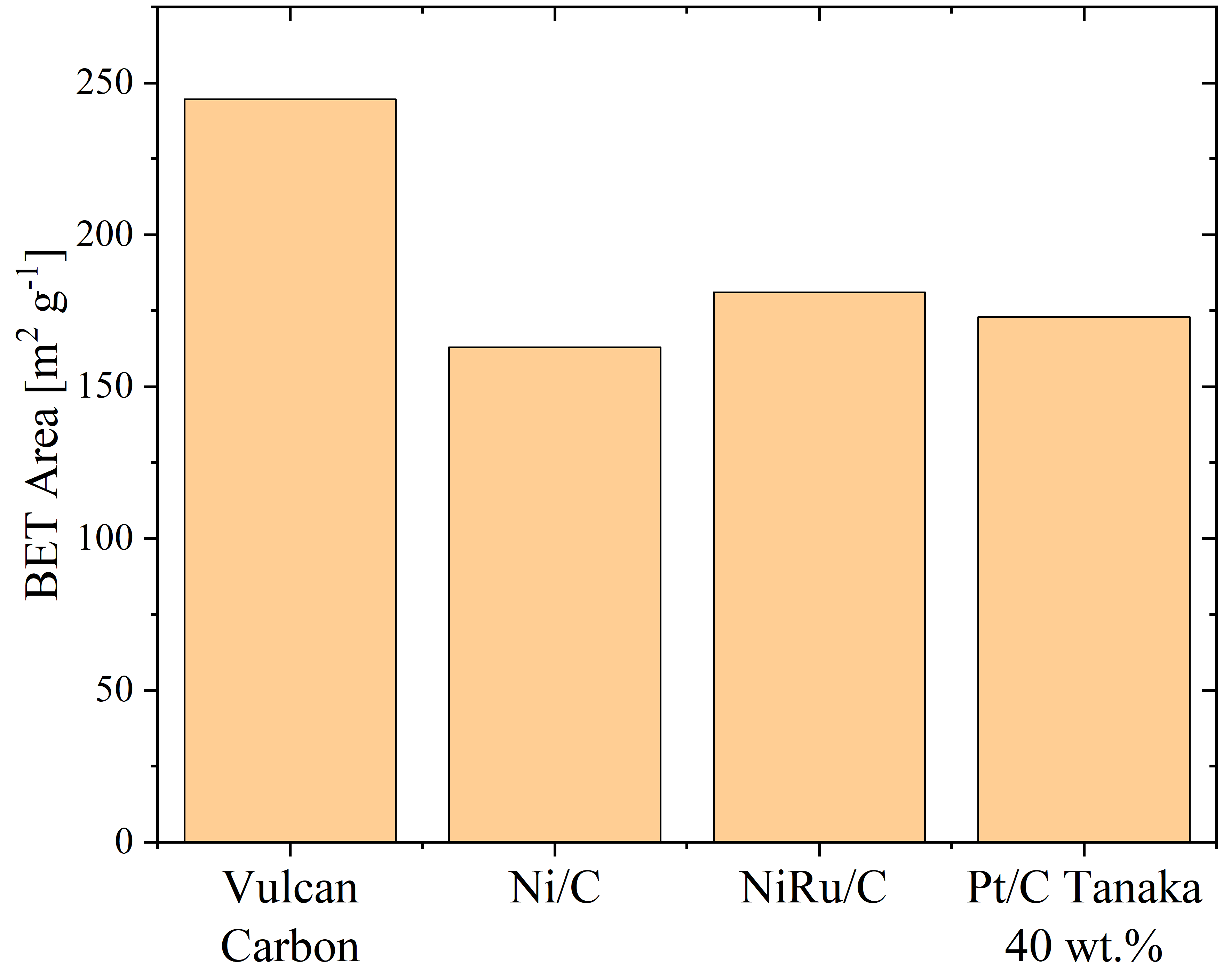


**Figure S8:** N_2_ adsorption/desorption experiments to determine the BET surface area of different catalyst materials. Vulcan carbon shows the highest BET surface area which decreases slightly when particles are attached onto the support material. This can be explained by the blocking of internal micro pores by the particles. ^[2]^


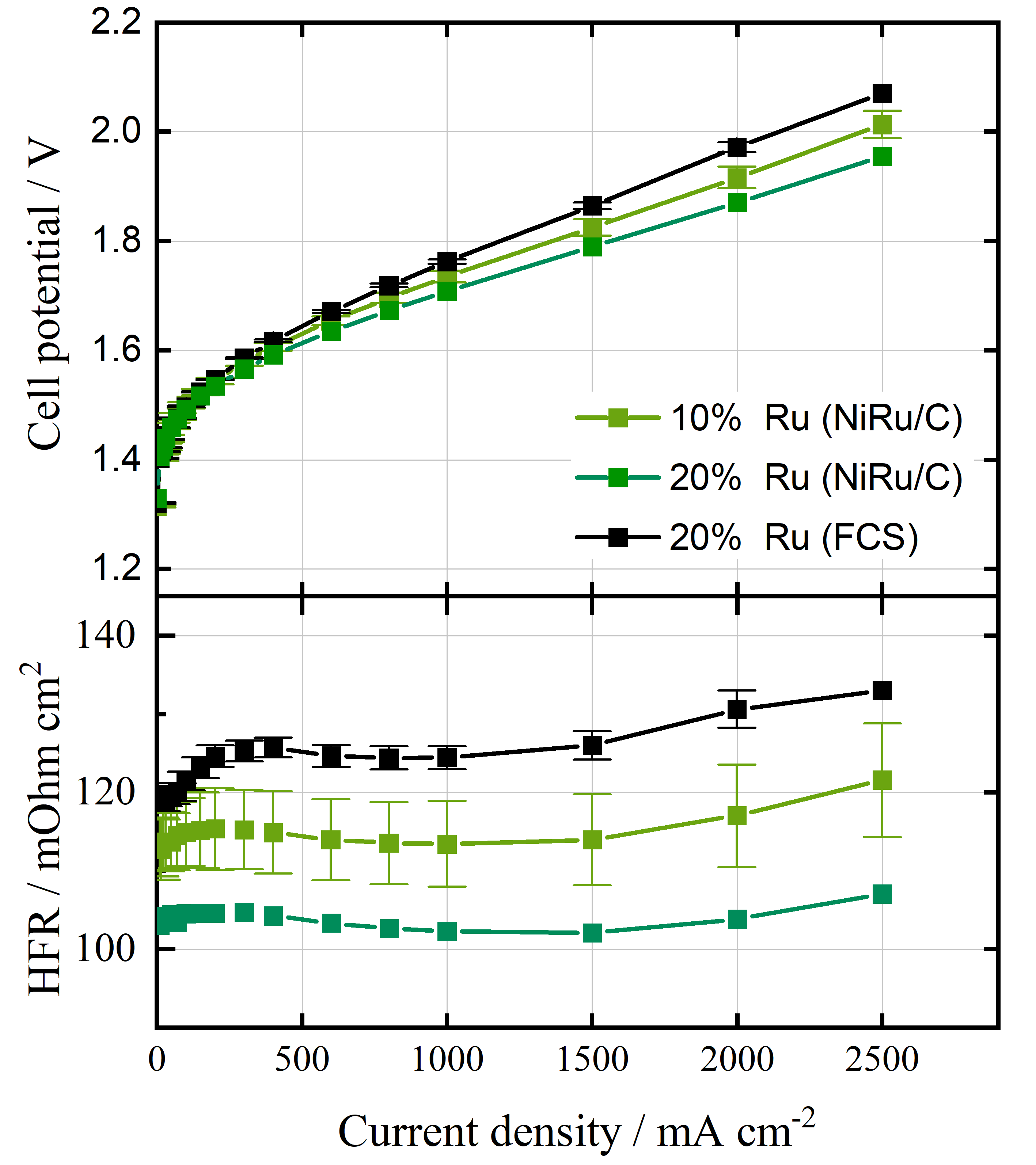


**Figure S9:** Polarization curves of the cells with 10 wt.% and 20 wt.% NiRu/C catalyst compared with 20 wt.% Ru/C reference (FCS)


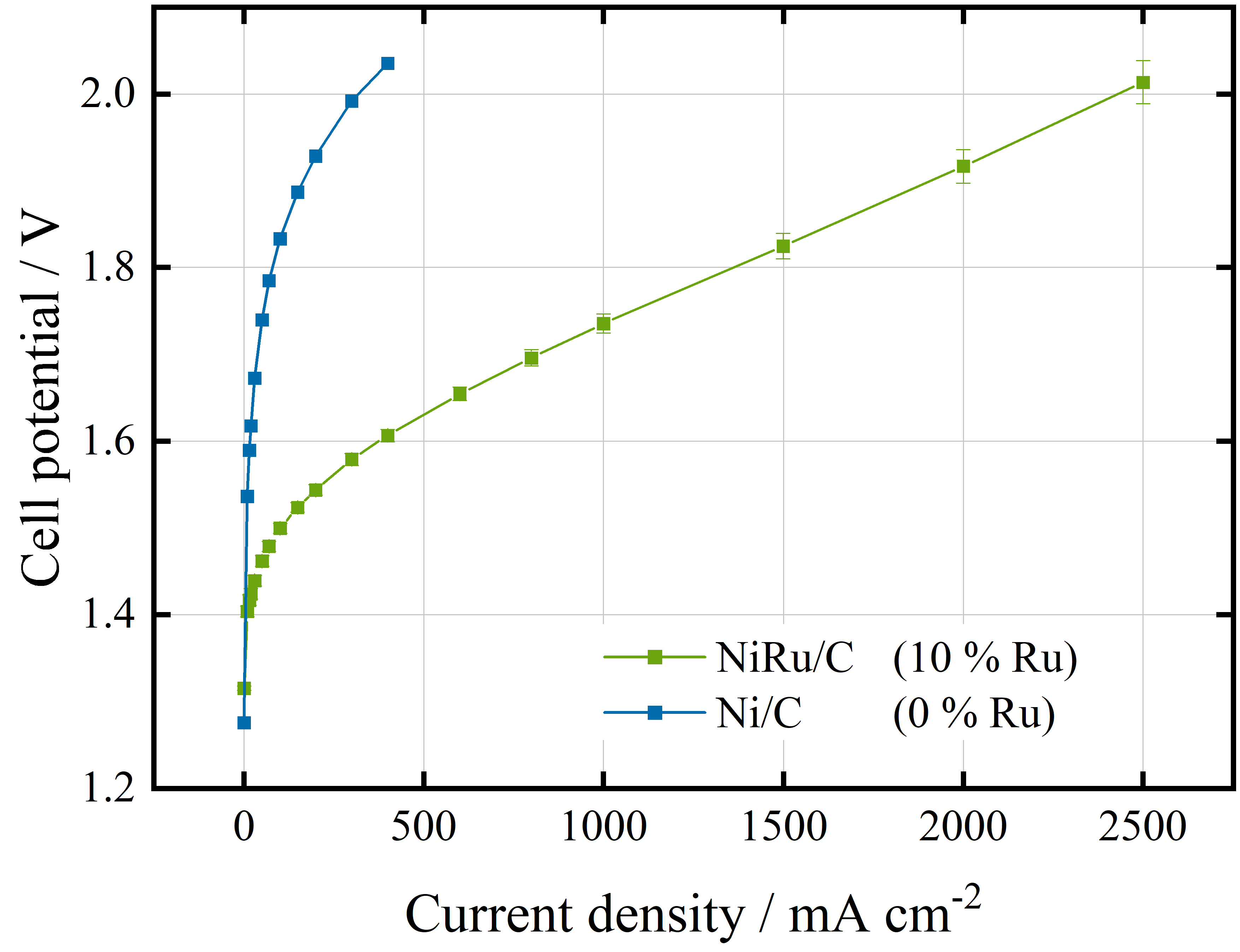


**Figure S10:** Polarization curves of the cells with Ni/C catalyst and NiRu/C. Ni/C catalyst shows very poor performance in full cell operation. The displacement of 10 wt.% Ru increases the performance of the material significantly

REFERENCES

[1] Pengxiang Song,a Dongsheng Wen, Z. X. Guob and Theodosios Korakianitis, Physical Chemistry Chemical Physics 2008, 5057.

[2] Rahman, M.M.; Inaba, K.; Batnyagt, G.; Saikawa, M.; Kato, Y.; Awata, R.; Delgertsetsega, B.; Kaneta, Y.; Higashi, K.; Uruga, T.; Iwasawa, Y.; Ui, K.; Takeguchi, T. *RSC Advances*, **2021**, *11,* 20601–20611.
